# Unraveling the impact of self-esteem on the utilization of Instagram filters: the mediating role of fear of negative evaluation

**DOI:** 10.3389/fpsyg.2024.1302662

**Published:** 2024-04-29

**Authors:** Calogero Lo Destro

**Affiliations:** Department of Psychology, Niccolò Cusano University, Rome, Italy

**Keywords:** self-esteem, fear of negative evaluation, Instagram filters, social media, photo-editing, digital alteration

## Abstract

The popularity of social media platforms, such as Instagram, has given rise to a surge in photo editing and filtering practices among users. Understanding the underlying psychological factors that drive individuals to employ filters is crucial in comprehending the impact of such behavior on self-perception and online interactions. This study aims to investigate the influence of self-esteem on the importance attributed to the use of Instagram filters, with a particular focus on the mediating role played by the fear of negative evaluation. A sample of participants (*N* = 2,657) from diverse demographic backgrounds has been recruited to complete a series of questionnaires measuring self-esteem, fear of negative evaluation, and Instagram filter usage patterns. Mediation analysis has been employed to assess the extent to which the fear of negative evaluation mediates the relationship between self-esteem and filter usage importance. In line with the hypotheses, the results revealed that participants self-esteem negatively predicted fear of negative evaluation, which in turn had a positive effect on use of filters. Furthermore, fear of negative evaluation fully mediated the relationship between self-esteem and use of filters. Overall, the implications of this research extend to both theoretical and practical domains. The findings can contribute to the existing literature on self-esteem, social media behavior, and body image by shedding light on the factors influencing digital self-presentation.

## 1 Introduction

The emergence of social network sites (SNSs) has engendered numerous novel opportunities for social interactions, owing to their highly conducive environment for self-presentation and establishing social connections. Compared to conventional media channels such as television and fashion magazines, social media platforms predominantly rely on content generated by peers. Thus, individuals engaged in social media are no longer subjected to exposure to the visually idealized representations of models featured in traditional magazines. Consequently, social media platforms should reflect a broad spectrum of typical body sizes and shapes exhibited by ordinary individuals (Tiggemann, 2022). However, the actual reality starkly contradicts this theoretical hypothesis. In fact, a cursory examination of nearly any social media platform reveals a landscape predominantly populated by individuals who, in contrast to the audience, possess exceptionally attractive physical attributes.

The current situation is shaped by the rise of social networking sites (SNS) and advanced technology. Individuals can now enhance their photos in real-time and during post-production. Instagram, especially, features built-in filters, displaying many photos of individuals with seemingly perfect physiques and facial attributes. The trend of photo editing poses monitoring challenges and precise statistics remain elusive. Recent research (Agrawal and Agrawal, 2021) reported that, at least, 25% of Instagram users edit over 40% of their shared content. However, this may be underestimated due to potential reluctance to acknowledge such behavior. Scholarly investigations (Chua and Chang, 2016; Dumas et al., 2017) have revealed that many women and girls invest significant time and effort in capturing and curating their self-portraits, with a practice of capturing 2–5 self-portraits before selecting the most suitable one. In photo modification, filters emerge as the most widely favored editing tool (Bij de Vaate et al., 2018).

The discrepancy theory (Higgins, 1987) posits that individuals are motivated to reduce the discrepancy between their actual and their ideal selves. According to this theory, when there’s a gap between the current and the ideal or desired state, people experience dissatisfaction or discomfort, which motivates them to take action to close such gap. Research in this field (Moretti and Higgins, 1990) has reported that the greater the disparity between individuals’ actual and ideal self, the lower their self-worth tends to be. In line with these assumptions, a discrepancy in appearance between one’s actual and ideal selves leads to low self-esteem (Jung et al., 2001). Notably, a significant relationship has been also identified between discrepancies in actual-ideal self-perceptions and fear of negative evaluation across various domains, such as in the context of public speaking tasks (Rodebaugh and Donahue, 2007). Furthermore, the fear of negative evaluation from others could exacerbate the impact of self-discrepancy on negative emotions such as shame and guilt (Liss et al., 2013).

As mentioned, discrepancy theory (Higgins, 1987) emphasizes the importance of self-regulation and the pursuit of congruence to reduce such discrepancies. Furthermore, it posits that individuals strive to align their behaviors and outcomes with their ideal self, which may be strongly related to the prevailing sociocultural standards. In the realm of social media, photo editing represents a process that facilitates potential adjustments through image manipulation to better align with this perceived ideal of beauty (Grogan et al., 2018). Research (Cohen et al., 2021; Tiggemann, 2022) suggest a potential negative association between photo editing practices on social networking sites (SNSs) and individuals’ levels of body satisfaction. Furthermore, users who engage in the practice of retouching their images have reported experiencing reduced feelings of attractiveness (Mills et al., 2018), and heightened negative mood and facial dissatisfaction (Tiggemann et al., 2020). While these studies have focused on the consequences of photo editing behaviors, few have attempted to explore their antecedents. The aim of this paper is to contribute to this field by investigating the role of self-esteem and fear of negative evaluation.

Self-esteem has been defined as an individual’s comprehensive assessment resulting in either a positive or negative orientation toward oneself (Rosenberg, 1965). Accordingly, individuals possessing high self-esteem demonstrate favorable evaluations of their self-concept, marked by a sense of admiration and value. Conversely, individuals with low self-esteem exhibit a dearth of self-approval, marked by sensations of insufficiency, unworthiness, and deficiency (Rosenberg, 1965). Individuals with low self-esteem are more inclined to internalize societal images of thinness (Striegel-Moore and Cachelin, 1999) and they tend to lean toward comparing their physical appearance more frequently (Van den Berg et al., 2007), seeking validation and support. Moreover, they are also more inclined to engage in upward comparisons (Perloff, 2014), which may serve as a reminder of the beauty standards they perceive themselves as not meeting (Patrick et al., 2004). As a consequence, individuals reporting low self-esteem tended to display higher self-promotion in their main profile picture (Mehdizadeh, 2010) and they were more incline to remove tags from unflattering pictures online (Tazghini and Siedlecki, 2013). In this regard, research (Michikyan et al., 2015) has also shown that individuals insecure about themselves may be more prone to engage in false self-presentation behaviors on Facebook, hoping to receive feedback that could bolster their perception of themselves.

Fear of negative evaluation is defined as a personal trait encompassing the concern and distress individuals endure in response to evaluations rendered by others, along with the desire to seek social approval while avoiding disapproval (Latimer and Martin Ginis, 2005). Self-esteem seems closely intertwined with fear of negative evaluation. In fact, assessing oneself negatively can lead to anticipating negative evaluations from others as well (Leary and Kowalski, 1995). In particular, research has shown that individuals with low self-esteem experienced increased fear of negative evaluation (Cheng et al., 2015). Further research (Kocovski and Endler, 2000) confirmed that low self-esteem predicted heightened fear of negative evaluation, with the latter, in turn, predicting increased social anxiety., A recent work (Naidu et al., 2023) has provided evidence supporting the assertion that young adults who experience fear about negative evaluation, triggered by their low self-esteem, tend to manifest both increased internet and social network problematic use. More importantly, research (Bodroža et al., 2022) has demonstrated that fear of negative evaluation positively predicted both self-presentation and selfie preoccupation, indicating that individuals who exhibited a stronger fear of negative appearance evaluation engaged more in self-presentation and were more preoccupied with taking selfies. The results of this study are particularly intriguing. Here, self-esteem and fear were both entered as predictors at the same level in a regression model. Surprisingly, self-esteem was found to be non-significant in predicting self-presentation. Fear of negative evaluation has also been found to act as a mediator in the relationship between contingent self-esteem and compulsive buying (Biolcati, 2017). Although these findings may not directly align with the current study’s focus, it has been reported (Dittmar et al., 1996) that items linked to appearance (such as clothes, shoes, and status symbol objects), often serve as symbols in the process of self-completion and self-promotion, conveying aspects of an individual’s identity to others. We postulate that a similar process may occur among individuals who edit their photos in an attempt to present themselves as better than their true selves, conforming to the ideal standards of beauty promoted by social media.

### 1.1 Hypotheses

Based on these assumptions, it is hypothesized that self-esteem negatively predicts the perceived importance of using Instagram filters. Additionally, given the significant negative relationship between self-esteem and the fear of negative evaluation, it is proposed that fear of negative evaluation may mediate the relationship between self-esteem and the importance of using filters. This is because photo editing could serve as a method for seeking social validation and mitigating fear related to potential negative evaluations from others.

## 2 Method

### 2.1 Participants

A total of 2,657 participants (1,864 females and 793 males), voluntarily took part in this research. Participants had a mean age of 21.91 (SD = 3.36). The mean age of males was 22.15 (SD 3.63), while the mean age of females was 21.81 (SD 3.24). 99.2% of the participants were Italian residents at the time of completing the test. Among the participants, 2.4% held a post-graduate specialization, 17.7% held a university degree, 72.8% held a high school degree, and 7.1% held a middle school diploma. Participants were provided with comprehensive information regarding the study and granted informed consent for the anonymous utilization of their data. They willingly completed a web-mediated survey and did not receive any form of remuneration for their participation. The study was carried out in compliance with the principles outlined in the Declaration of Helsinki.

### 2.2 Procedures

The survey consisted of an initial part that included multiple questions pertaining to socio-demographic information. Specifically, respondents were asked about their age, gender, country of residence, and level of education attained from 1 (Elementary License) to 5 (Postgraduate Specialization/PhD/Other). Research has revealed that gender and age are implicated in individuals’ tendency to edit photos, with women and younger people being more active in this area (Dhir et al., 2016). Similarly, it has been found that social media use at time 1 indirectly increased the frequency of selfie editing at time 2 (Chae, 2017). Furthermore, in line with the procedures of previous studies (Mills et al., 2018, p. 201; Beos et al., 2021), participants were also asked to report the number of followers associated with their personal profiles. To evaluate self-esteem, the well-established Rosenberg Self-Esteem Scale (Rosenberg, 1965) was utilized. In addition, participants were required to provide their responses to a question specifically designed to assess the perceived importance of applying filters to their photos on Instagram. The mediator variable in this study was fear of negative evaluation.

### 2.3 Materials

The Rosenberg Self-Esteem Scale consists of a total of 10 items. Among these items, six are formulated in a positive manner (e.g., items 1, 2, 4, 6, 7, and 8), while the remaining four are formulated in a negative manner (e.g., items 3, 5, 9, and 10). For example, a positive item is “I feel that I possess several positive qualities,” whereas a negative item is “I tend to believe that I am a failure.” Participants were instructed to indicate their responses on a 4-point Likert scale, ranging from 0 (strongly disagree) to 3 (strongly agree). To ensure uniformity in scoring, the responses to the negatively worded items were reversed prior to data analysis. The reliability of the scale was evaluated using Cronbach’s alpha coefficient, which yielded a value of 0.85 in this study.

Over the years, various shortened adaptations of the Brief Fear of Negative Evaluation Scale (BFNE) have been utilized (Roberts et al., 2014; Perczel-Forintos and Kresznerits, 2017). In the current research, a version composed by five (i.e., “I am frequently afraid of other people noticing my shortcomings.”; “I am afraid that others will not approve of me.”; “When I am talking to someone, I worry about what they may be thinking about me.”; “Sometimes I think I am too concerned with what other people think of me.”; “I often worry that I will say or do the wrong things.”) of the original 12 items was adopted. To calculate the overall scores for these items, answers on the five items were averaged. Participants provided ratings for each item on a 5-point Likert scale, where scores ranged from 1 (Not at all) to 5 (Extremely). The sample’s Cronbach’s alpha coefficient was 0.88.

Instagram filters importance was assessed by asking participants to provide their response to a customized question, namely, “How important is it to apply filters to your photos to enhance lighting, shadows, and colors before posting?” This question was specifically designed to measure the importance placed on photo editing practices on the Instagram platform. Participants were asked to indicate their response to the aforementioned question using a 4-point Likert scale, which ranged from 0 “Not important (I never edit photos)” to 3 “It is of fundamental importance (I never post a “natural” photo).”

## 3 Results

The assumption of normality seems to be satisfied for the main variable of interest in the study. To elaborate, the kurtosis and skewness values for the importance attributed to filters were −0.125 and 0.280, respectively. Likewise, for self-esteem, the corresponding kurtosis and skewness values were −0.177 and −0.233, and for fear of negative evaluation, they were −0.304 and 0.436, respectively. Table 1 presents descriptive statistics and correlations among the variables. The results revealed a significant positive correlation between fear of negative evaluation and the importance attributed to photo filters. Conversely, self-esteem exhibited a negative correlation with both fear of negative evaluation and the importance placed on photo filters. Moreover, gender was significantly correlated with importance attributed to filters, indicating that females tended to attribute higher importance to the use of filters compared to males. Additionally, the importance of photo filters showed a positive correlation with the amount of time spent on Instagram. Lastly, fear of negative evaluation exhibited a negative relationship with age, suggesting that as age increased, fear of negative evaluation tended to decrease.

**Table 1:** Correlations between variables.

	*M* (SD)	1	2	3	4	5	6	7
1. Gender	– (−)	(−)						
2. Age	21.91 (3.36)	−0.05*						
3. Daily hours Instagram	3.30 (1.00)	0.08**	−0.11**					
4. Number of followers	1,029 (2,517)	0.03	−0.05*	0.10**				
5. Self-esteem	19.56 (5.24)	−0.06**	0.07**	−0.14**	0.02			
6. FNE	2.54 (0.88)	0.05*	−0.10**	0.07**	0.003	−0.53**		
7. Instagram filters use	1.37 (0.73)	0.24**	−0.04	0.14**	0.11**	−0.09*	0.17**	(−)

*p < 0.05, **p < 0.01; *N* = 2,657.

To examine the hypothesis that fear of negative evaluation mediated the association between self-esteem and the importance attributed to the use of filters, the PROCESS macro Model 4 (Hayes, 2013) was employed. This statistical tool utilizes the bootstrapping method to generate estimates of both direct and indirect effects. More specifically, a 95% confidence interval alongside a bootstrap method with 5,000 iterations has been adopted. Predictor variables were centered prior to analysis. Control variables, including gender (with male coded as 0 and female coded as 1), age, education, daily hours spent on Instagram, and the number of followers on participants’ Instagram profiles, were included in the analysis. A summary of the results is reported in Table 2.

**Table 2:** Summary of the regression models.

	Fear of negative evaluation				Instagram filters use			
	ß	SE	*p*-value	CI	ß	SE	*p*-value	CI
Gender	0.02	0.03	0.344	−0.03; 0.09	0.22	0.03	0.000	0.29; 0.41
Age	−0.06	0.004	0.001	−0.02; −0.01	0.003	0.004	0.871	−0.01; 0.01
Daily hours Instagram	−0.01	0.01	0.627	−0.04; 0.02	0.11	0.01	0.000	0.05; 0.11
Number of followers	0.01	0.00	0.415	0.00; 0.00	0.09	0.001	0.000	0.00; 0.00
Self-esteem	−0.53	0.003	0.000	−0.09; −0.08	0.02	0.003	0.342	−0.003; 0.01
Fear of negative evaluation					0.16	0.02	0.000	0.10; 0.17
*R*^2^	0.29				0.10			

*N* = 2,657.

The findings indicated a negative and significant impact of self-esteem on fear of negative evaluation. Additionally, age negatively predicted fear of negative evaluation, indicating that younger individuals reported higher levels of fear. This model was significant with an *R*^2^ of 0.29 [*F* (5, 2,651) = 216.67, *p* < 0.001].

**Figure 1:**
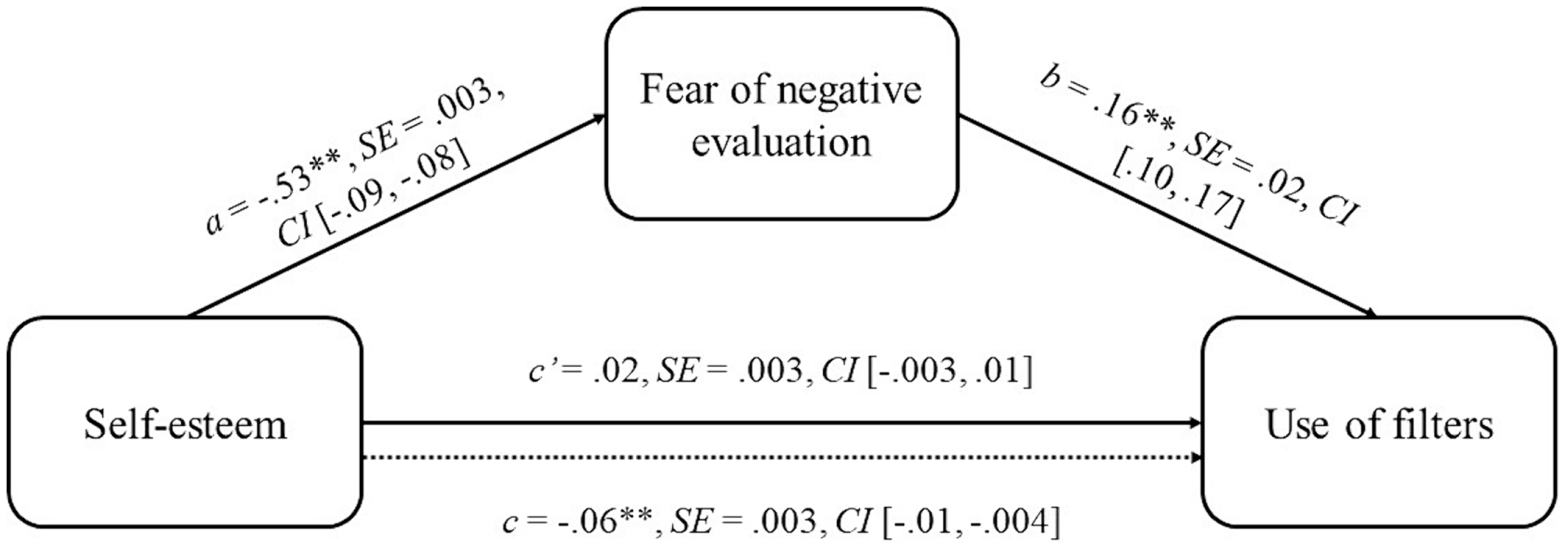
Mediation model. *p < 0.05; **p < 0.01. Standardized regression coefficients are reported. Dotted line denotes the effect of self-esteem on use of filters, when fear of negative evaluation is not included as a mediator.

Importantly, fear of negative evaluation demonstrated a significant and positive influence on the importance attributed to the use of filters. Furthermore, gender positively predicted importance attributed to filters, with women tending to assign greater importance; Additionally, both the amount of time spent on Instagram and the number of followers positively predicted importance attributed to the use of photo filters. Notably, the overall model was significant with an *R*^2^ of 0.10 [*F* (6, 2,650) = 49.78, *p* < 0.001]. Furthermore, upon controlling for fear of negative evaluation, the association between self-esteem and the importance of using filters became statistically non-significant, suggesting a complete mediation effect (see Figure 1). Accordingly, the indirect effect of self-esteem on the importance attributed to the use of filters through fear of negative evaluation was significant (*β* = −0.08, BootSE = 0.01; bootstrapping CI = [−0.11, −0.06]). The total effect of self-esteem on Instagram use of filters was significant (*ß* = −0.06, SE = 0.003, *p* = 0.001, CI = [−0.01, −0.004]).

## 4 Discussion

The extensive practice of digitally manipulating photos is acknowledged as one of primary factors contributing to the widespread distortion of reality observed on social media platforms (Nightingale et al., 2017; Shen et al., 2019). Prominent social networking platforms, such as Instagram, incorporate inherent filters designed to manipulate photographic attributes. The ubiquity of these digital manipulation tools within the realm of social media significantly contributes to the presentation of idealized and implausible self-representations on the digital platform (Naderer et al., 2021). In this vein, the primary objective of this investigation was to scrutinize the influence of two variables that could potentially either dissuade or compel individuals to partake in heightened levels of image manipulation on social media platforms. According to the assumptions of the discrepancy theory (Higgins, 1987), the present study postulates that self-esteem and fear of negative evaluation represent two relevant antecedents shaping such behavioral patterns.

In line with the hypotheses, this research demonstrated that self-esteem was negatively, although not directly, related to importance attributed to the use of filters and negatively associated with fear of negative evaluation. Furthermore, it has been assumed that particular socio-demographic and Instagram-associated factors may play a substantial role in shaping importance attributed to photo filters. To be specific, as previously reported (Willemse et al., 2023), age emerged as a predictive determinant of fear of negative evaluation, wherein individuals of a younger age demonstrated elevated levels of fear of negative evaluation relative to their older counterparts. Although gender, daily time spent on Instagram, and the count of followers did not yield statistically significant predictions for the fear of negative evaluation, they did manifest a noteworthy positive influence on the perceived importance assigned to the use of Instagram filters. Notably, females exhibited a heightened propensity for attributing greater importance to filters. This finding appears congruent with earlier research within this domain, which delineated a stronger tendency among females toward engaging in photo manipulation (Toh et al., 2022). Similarly, the extent of daily engagement with Instagram and the magnitude of one’s follower count contribute to amplify the importance attributed to Instagram photo editing. Consistent with earlier research on the subject (Dhir et al., 2016), younger users have been found to exhibit greater engagement in the practice of photo editing.

Importantly, the study found that fear of negative evaluation mediated the relationship between self-esteem and importance attributed to photo editing. This means that the negative relationship between self-esteem and fear of negative evaluation partially explains why people with a positive image of themself are less likely to attribute importance to photo editing and use of filters. In other words, individuals characterized by elevated self-esteem tend to manifest heightened self-assuredness, thereby attributing lesser significance to external opinions. Consequently, this diminished susceptibility to the fear of negative evaluation contributes to their reduced propensity to bestow heightened importance upon Instagram filters. While this pattern share similarities with that explored in prior research (Biolcati, 2017), it is important to note distinctions in the dependent variables under investigation: compulsive buying versus photo editing. Previous research (Bodroža et al., 2022) has additionally demonstrated that when self-esteem and fear of negative evaluation were simultaneously included into a regression model, the predictive effect of fear of negative evaluation on self-presentation was negative and significant, whereas the effect of self-esteem was not significant. However, the measure of self-presentation used in the study by Bodroža et al. (2022) was not directly associated with the editing dimension. Hence, the findings of the current study, in accordance with the discrepancy theory (Higgins, 1987), contribute to elucidating the relationship between self-esteem, fear of negative evaluation, and the significance attributed to filters within the realms of social media.

It is also important to acknowledge the limitations of the present research. Foremost among these limitations is the sole utilization of self-report instruments, which introduces the potential for common method or source bias. Furthermore, for the assessment of the importance attributed to the use of Instagram filters, a standardized test was not employed; instead, an *ad-hoc* question was adopted. While this approach served the specific objectives of the research, it introduces psychometric limitations. In addition, other potential contributing factors to importance attributed to filters such as peer influence, media and celebrity influence or online identity and branding have not been accounted for in this study. Additionally, the lack of information about the type of activity participants do on social media should be addressed by future research. Other than this, this study has not accounted for the specific genre of photographs, such as selfies, to assess potential variations in the effects of importance attributed to filters, as compared to other categories of images, such as landscapes. Another potential limitation to consider is the possibility that the relationship between self-esteem and photo alteration may not be unidirectional; it could be circular in nature. In other words, modifying one’s photos might lead to an enhanced self-perception, thereby reinforcing self-esteem. This intricate interplay warrants further investigation through longitudinal studies to gain a deeper understanding of this dynamic association.

From a practical perspective, the findings of this research have relevance for digital media practitioners, social media platform designers, and educators. Understanding the influence of self-esteem and its interplay with the fear of negative evaluation in driving the use of Instagram filters can inform the development of strategies to promote healthier online behaviors and cultivate a more positive digital environment. Additionally, digital media literacy programs can incorporate these insights to empower users to engage with social media platforms mindfully and critically, ensuring a balanced and authentic presentation of themselves online. Through such applications, this research aims to contribute to the cultivation of a more informed and responsible digital world.

In conclusion, the primary objective of this study was to elucidate the underlying factors influencing the alteration of photographs on Instagram. More specifically, this research endeavored to establish that self-esteem and fear of negative evaluation, potentially constitutes two fundamental dimensions intricately implicated in the decision to edit or abstain from modifying content shared on social media platforms. Results suggest that self-esteem, through fear of negative evaluation, potentially plays a mitigating role in preventing individuals from attributing importance to the act of photo editing on Instagram. Conversely, the fear of negative evaluation emerges as a potential risk factor, predisposing individuals to ascribe heightened importance to this particular practice. Future research holds promise in investigating the multifaceted aspects involved in the potential negative outcomes associated with the use of social media platforms.

## Data availability statement

The raw data supporting the conclusions of this article will be made available by the authors, without undue reservation.

## Ethics statement

The studies involving humans were approved by Research Board Committee - Niccolò Cusano University of Rome. The studies were conducted in accordance with the local legislation and institutional requirements. The participants provided their written informed consent to participate in this study.

## Author contributions

CL: Writing – original draft, Writing – review & editing.
