# Supplementary material for: Unraveling the impact of self-esteem on the utilization of Instagram filters: the mediating role of fear of negative evaluation
Source: Front Psychol. 2024 Apr 29;15:1302662. doi: 10.3389/fpsyg.2024.1302662 (PMC11092376; doi:10.3389/fpsyg.2024.1302662)
Supplement: Supplementary file 1 [file Table_1.DOCX]

Run MATRIX procedure:

***************** PROCESS Procedure for SPSS Version 3.5 *****************

Written by Andrew F. Hayes, Ph.D. www.afhayes.com

Documentation available in Hayes (2018). www.guilford.com/p/hayes3

**************************************************************************

Model : 4

Y : filtri

X : ESTEEM

M : BFNE

Covariates:

studio follow ore_ig Età

Sample

Size: 793

**************************************************************************

OUTCOME VARIABLE:

BFNE

Model Summary

R R-sq MSE F df1 df2 p

,5493 ,3017 ,5160 68,0092 5,0000 787,0000 ,0000

Model

coeff se t p LLCI ULCI

constant 4,5668 ,2320 19,6872 ,0000 4,1115 5,0222

ESTEEM -,0924 ,0052 -17,8654 ,0000 -,1025 -,0822

studio ,0509 ,0486 1,0480 ,2950 -,0445 ,1464

follow ,0000 ,0000 -1,1970 ,2317 -,0001 ,0000

ore_ig -,0039 ,0270 -,1444 ,8852 -,0569 ,0491

Età -,0166 ,0077 -2,1597 ,0311 -,0317 -,0015

Standardized coefficients

coeff

ESTEEM -,5419

studio ,0342

follow -,0360

ore_ig -,0044

Età -,0704

**************************************************************************

OUTCOME VARIABLE:

filtri

Model Summary

R R-sq MSE F df1 df2 p

,2305 ,0531 ,4475 7,3486 6,0000 786,0000 ,0000

Model

coeff se t p LLCI ULCI

constant ,3997 ,2639 1,5144 ,1303 -,1184 ,9177

ESTEEM -,0005 ,0057 -,0813 ,9353 -,0117 ,0107

BFNE ,1127 ,0332 3,3964 ,0007 ,0476 ,1779

studio ,0867 ,0453 1,9149 ,0559 -,0022 ,1756

follow ,0001 ,0000 2,9840 ,0029 ,0000 ,0001

ore_ig ,0812 ,0252 3,2289 ,0013 ,0318 ,1306

Età -,0068 ,0072 -,9424 ,3463 -,0209 ,0073

Standardized coefficients

coeff

ESTEEM -,0034

BFNE ,1411

studio ,0728

follow ,1047

ore_ig ,1152

Età -,0359

************************** TOTAL EFFECT MODEL ****************************

OUTCOME VARIABLE:

filtri

Model Summary

R R-sq MSE F df1 df2 p

,1980 ,0392 ,4535 6,4252 5,0000 787,0000 ,0000

Model

coeff se t p LLCI ULCI

constant ,9146 ,2175 4,2056 ,0000 ,4877 1,3414

ESTEEM -,0109 ,0048 -2,2443 ,0251 -,0204 -,0014

studio ,0925 ,0456 2,0295 ,0427 ,0030 ,1819

follow ,0001 ,0000 2,8230 ,0049 ,0000 ,0001

ore_ig ,0808 ,0253 3,1902 ,0015 ,0311 ,1305

Età -,0086 ,0072 -1,1986 ,2310 -,0228 ,0055

Standardized coefficients

coeff

ESTEEM -,0799

studio ,0776

follow ,0997

ore_ig ,1146

Età -,0458

************** TOTAL, DIRECT, AND INDIRECT EFFECTS OF X ON Y **************

Total effect of X on Y

Effect se t p LLCI ULCI c_ps c_cs

-,0109 ,0048 -2,2443 ,0251 -,0204 -,0014 -,0159 -,0799

Direct effect of X on Y

Effect se t p LLCI ULCI c'_ps c'_cs

-,0005 ,0057 -,0813 ,9353 -,0117 ,0107 -,0007 -,0034

Indirect effect(s) of X on Y:

Effect BootSE BootLLCI BootULCI

BFNE -,0104 ,0031 -,0165 -,0046

Partially standardized indirect effect(s) of X on Y:

Effect BootSE BootLLCI BootULCI

BFNE -,0152 ,0044 -,0240 -,0067

Completely standardized indirect effect(s) of X on Y:

Effect BootSE BootLLCI BootULCI

BFNE -,0765 ,0225 -,1208 -,0337

*********************** ANALYSIS NOTES AND ERRORS ************************

Level of confidence for all confidence intervals in output:

95,0000

Number of bootstrap samples for percentile bootstrap confidence intervals:

5000

------ END MATRIX -----

Run MATRIX procedure:

***************** PROCESS Procedure for SPSS Version 3.5 *****************

Written by Andrew F. Hayes, Ph.D. www.afhayes.com

Documentation available in Hayes (2018). www.guilford.com/p/hayes3

**************************************************************************

Model : 4

Y : filtri

X : ESTEEM

M : BFNE

Covariates:

studio follow ore_ig Età

Sample

Size: 1864

**************************************************************************

OUTCOME VARIABLE:

BFNE

Model Summary

R R-sq MSE F df1 df2 p

,5400 ,2916 ,5565 152,9391 5,0000 1858,0000 ,0000

Model

coeff se t p LLCI ULCI

constant 4,4186 ,1579 27,9900 ,0000 4,1090 4,7282

ESTEEM -,0891 ,0033 -27,0367 ,0000 -,0956 -,0826

studio ,1474 ,0341 4,3211 ,0000 ,0805 ,2144

follow ,0000 ,0000 1,1001 ,2714 ,0000 ,0000

ore_ig -,0066 ,0175 -,3751 ,7076 -,0408 ,0277

Età -,0265 ,0059 -4,4630 ,0000 -,0382 -,0149

Standardized coefficients

coeff

ESTEEM -,5357

studio ,0939

follow ,0216

ore_ig -,0074

Età -,0970

**************************************************************************

OUTCOME VARIABLE:

filtri

Model Summary

R R-sq MSE F df1 df2 p

,2309 ,0533 ,4851 17,4241 6,0000 1857,0000 ,0000

Model

coeff se t p LLCI ULCI

constant ,6353 ,1758 3,6150 ,0003 ,2906 ,9800

ESTEEM ,0025 ,0036 ,6951 ,4871 -,0046 ,0096

BFNE ,1323 ,0217 6,1084 ,0000 ,0898 ,1748

studio ,0869 ,0320 2,7147 ,0067 ,0241 ,1497

follow ,0000 ,0000 4,1328 ,0000 ,0000 ,0000

ore_ig ,0784 ,0163 4,8047 ,0000 ,0464 ,1104

Età -,0045 ,0056 -,8147 ,4154 -,0155 ,0064

Standardized coefficients

coeff

ESTEEM ,0188

BFNE ,1639

studio ,0686

follow ,0940

ore_ig ,1103

Età -,0206

************************** TOTAL EFFECT MODEL ****************************

OUTCOME VARIABLE:

filtri

Model Summary

R R-sq MSE F df1 df2 p

,1851 ,0343 ,4946 13,1886 5,0000 1858,0000 ,0000

Model

coeff se t p LLCI ULCI

constant 1,2200 ,1488 8,1971 ,0000 ,9281 1,5119

ESTEEM -,0093 ,0031 -2,9820 ,0029 -,0154 -,0032

studio ,1064 ,0322 3,3085 ,0010 ,0433 ,1695

follow ,0000 ,0000 4,2487 ,0000 ,0000 ,0000

ore_ig ,0775 ,0165 4,7060 ,0000 ,0452 ,1098

Età -,0081 ,0056 -1,4375 ,1507 -,0190 ,0029

Standardized coefficients

coeff

ESTEEM -,0690

studio ,0840

follow ,0975

ore_ig ,1091

Età -,0365

************** TOTAL, DIRECT, AND INDIRECT EFFECTS OF X ON Y **************

Total effect of X on Y

Effect se t p LLCI ULCI c_ps c_cs

-,0093 ,0031 -2,9820 ,0029 -,0154 -,0032 -,0130 -,0690

Direct effect of X on Y

Effect se t p LLCI ULCI c'_ps c'_cs

,0025 ,0036 ,6951 ,4871 -,0046 ,0096 ,0035 ,0188

Indirect effect(s) of X on Y:

Effect BootSE BootLLCI BootULCI

BFNE -,0118 ,0021 -,0161 -,0078

Partially standardized indirect effect(s) of X on Y:

Effect BootSE BootLLCI BootULCI

BFNE -,0165 ,0029 -,0224 -,0111

Completely standardized indirect effect(s) of X on Y:

Effect BootSE BootLLCI BootULCI

BFNE -,0878 ,0155 -,1197 -,0587

*********************** ANALYSIS NOTES AND ERRORS ************************

Level of confidence for all confidence intervals in output:

95,0000

Number of bootstrap samples for percentile bootstrap confidence intervals:

5000

------ END MATRIX -----
